# Therapeutic Potential of Niacin in PS19 Tauopathy Mice

**DOI:** 10.1002/alz70855_102381

**Published:** 2025-12-23

**Authors:** Israel Coronel, Henika Patel, Abigail Wallace, Disha Soni, Pablo Martinez, Claudia Rangel‐Barajas, Adrian L Oblak, Cristian A Lasagna Reeves, Gary E Landreth, Miguel Moutinho

**Affiliations:** ^1^ Stark Neurosciences Research Institute, Indiana University School of Medicine, Indianapolis, IN, USA; ^2^ Indiana University School of Medicine, Indianapolis, IN, USA

## Abstract

**Background:**

Alzheimer's disease (AD) is characterized by the presence of extracellular amyloid‐b plaques, intraneuronal neurofibrillary tangles, and a robust immune response. Dietary intake of niacin (nicotinic acid) has been correlated with decreased risk of AD and age‐related cognitive decline. We have recently shown that niacin stimulates the receptor HCAR2 to induce a protective microglial phenotype and attenuated disease severity in an amyloid mouse model of AD. However, the therapeutic potential of HCAR2 in tauopathy remains unknown.

**Methods:**

To investigate the contribution of HCAR2 on tau pathology, we used the tauopathy mouse model PS19. We employed two different strategies of niacin treatment, daily oral gavage (100 mg/kg) for 30 days between 9‐10 months of age, and a niacin‐enriched diet between 6‐9 months of age. To assess the effect of niacin on tau pathology, we analyzed motor phenotype, microglial and synaptic markers, as well as the levels of tau species in PS19 mice.

**Results:**

Our preliminary findings indicate that HCAR2 expression is significantly elevated in hippocampal microglia of PS19 mice. Oral niacin treatment ameliorates motor coordination deficits and prevents neuronal loss, suggesting a restoration of synaptic integrity. Furthermore, genetic deletion of the HCAR2 receptor in PS19 mice, accelerated the onset of motor deficits and exacerbated the accumulation of pathogenic species of tau. These results suggest that HCAR2 is protective in tau pathology and activating this receptor could serve as a promising pharmacological strategy to mitigate disease severity.

**Conclusion:**

Our findings suggest that alterations in hippocampal HCAR2 expression may play a role in tau pathology. Niacin treatment improved motor coordination but did not affect the clasping reflex. In addition, niacin improved expression patterns of structural synaptic proteins. These results indicate that HCAR2 activation may help reduce disease severity. However, further studies are needed to clarify the underlying mechanisms. Overall, this research highlights a novel role for HCAR2 in tauopathies and supports the potential repurposing of existing niacin formulations as a therapeutic approach for Alzheimer's disease.

